# Expanded Properties and Applications of Porous Flame-Retardant Polymers Containing Graphene and Its Derivatives

**DOI:** 10.3390/polym16142053

**Published:** 2024-07-18

**Authors:** Shan Liu, Min He, Qingdong Qin, Wei Liu, Longfeng Liao, Shuhao Qin

**Affiliations:** 1 College of Materials and Energy Engineering, Guizhou Institute of Technology, Guiyang 550003, China; 2 College of Materials and Metallurgy, Guizhou University, Guiyang 550025, China

**Keywords:** porous material, graphene, flame-retardancy, multiple energy conversion, thermal insulation, EMI shielding, adsorption

## Abstract

With the integration and miniaturization of modern equipment and devices, porous polymers, containing graphene and its derivatives, with flame-retardancy have become a research hotspot. In this paper, the expanded properties and high-end applications of flame-retardant porous materials containing graphene and its derivatives were discussed. The research progress regarding graphene-based porous materials with multiple energy conversion, thermal insulation, an electromagnetic shielding property, and a high adsorption capacity were elucidated in detail. The potential applications of materials with the above-mentioned properties in firefighter clothing, fire alarm sensors, flexible electronic skin, solar energy storage, energy-saving buildings, stealth materials, and separation were summarized. The construction strategies, preparation methods, comprehensive properties, and functionalization mechanisms of these materials were analyzed. The main challenges and prospects of flame-retardant porous materials containing graphene and its derivatives with expanded properties were also proposed.

## 1. Introduction

Graphene, a carbon material with a two-dimensional (2D), one-layer sheet structure, has drawn a lot of interest because of its distinct mechanical, electrical, and physicochemical characteristics [1,2]. Compared with carbon nanotubes (CNTs) or graphite, graphene is a new class of filler with exceptional functions. It is abundant in nature, and has an extremely high specific surface area, which enables transferring higher levels of stress across its interface and provides higher reinforcement than CNTs; it has isotropic electrical/thermal conductivities on the graphene plane; and it has a low viscosity and is nontoxic when compounded with polymers [3].

Graphene is reported to have a wide range of potential applications in energy storage, electronics, electrocatalysis, and sensors [4,5]. Due to the high dependence of macroscopic performance on the microstructure of graphene, researchers have precisely regulated its structure to prepare a series of materials that is suitable for their applications [6,7,8].

Typical graphene derivatives are graphene nanoplates (GNPs) [9], graphene oxide (GO) [10], reduced graphene oxide (rGO) [11], expanded graphite [12], fluorinated graphene, graphene aerogel (GA) [13], etc. Among them, GO is grafted with oxygen-containing functional groups like carboxyl, hydroxyl, and epoxy, while maintaining the 2D-layered structure of graphene. These functional groupings offer enough anchor points to reinforce the matrix–GO connection, so GO has been extensively utilized to enhance the overall performance of polymer matrices [14]. GAs are great candidates for energy storage, as well as absorbing media applications due to their exceptional electrical conductivity, as they exhibit significant specific surface area and porosity [15,16]. However, the stacking and agglomeration of graphene sheets will impair their advantages in terms of specific surface area and active adsorption sites [17]. Moreover, many GAs are constructed through electrostatic interactions, H-bonding, and π–π stacking, so their mechanical properties are poor and their porous structures are likely to collapse, which affects their application, durability, and recyclability [18,19].

With the miniaturization, integration, and intelligence of modern equipment and devices, the number of electronic components per unit area has increased exponentially. If the heat emitted by electronic components during operation cannot be dissipated in time, it will cause the components to overheat, directly affecting their lifespan, and, in severe cases, may cause a fire hazard. Effective thermal management and flame-retardant design need to be considered [20,21,22]. Compared with traditional flame retardants, graphene and its derivatives not only have high stability, but also high thermal conductivity, which is more conducive to heat dissipation and flame-retardancy.

Porous materials have many pore structures, which can reduce the density of the material and increase the surface area. By regulating the pore size and structure, these materials can be made to possess specially designed properties and expanded applications. The development of porous materials, containing graphene and its derivatives, that possess both flame-retardancy and other expanded properties has become a research hotspot [23,24]. Multifunctional features, combined with flame-retardancy, can be transformed into new applications, such as energy storage, separation and adsorption, thermal insulation, etc. [25,26,27].

In existing reviews, the possibility of the use of graphene as a flame retardant material for polymeric materials was evaluated, and the effect of graphene dispersion on the flame-retardancy of polymers as well as methods to improve its dispersion were also documented [28,29]. However, the demands and applications of graphene-based porous materials with flame-retardancy and expanded properties have never been systematically summarized.

**Figure 1:**
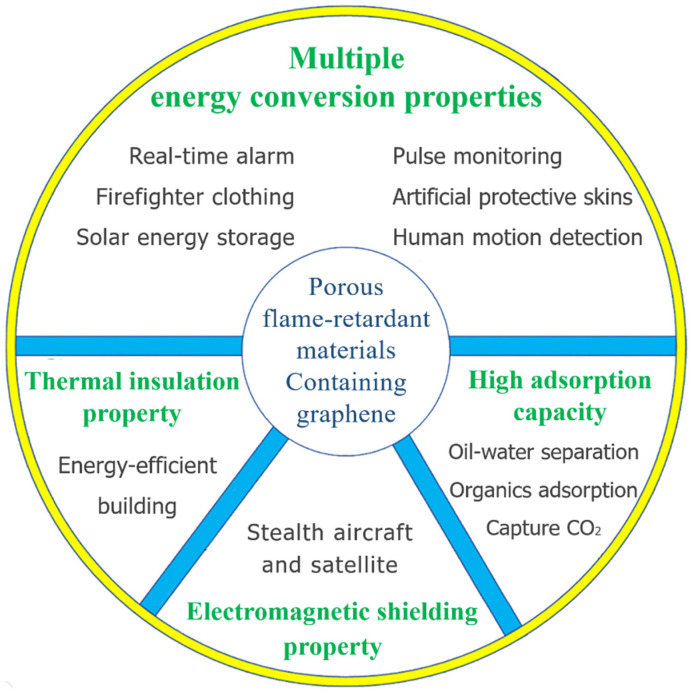
Expanded properties and related applications of porous flame-retardant materials containing graphene.

In this review, the expanded properties and related applications of porous flame-retardant materials containing graphene were summarized (Figure 1). The characteristics of graphene-based porous materials with expanded properties, including multiple energy conversion, thermal insulation, electromagnetic shielding property, and high adsorption capacity, were elucidated in detail. The potential applications of materials with the above-mentioned properties in firefighter clothing, fire alarm sensors, flexible electronic skin, solar energy storage, energy-saving buildings, stealth materials, and separation were summarized. The construction strategies, preparation methods, comprehensive properties, and functionalization mechanisms of these materials were analyzed. The main challenges and prospects regarding the use of graphene-based porous materials with expanded properties were also outlined.

## 2. Flame Retardant Porous Materials Containing Graphene and Its Derivatives

Highly heat-resistant graphene and its derivatives were proved to have promising flame-retardancy, which can reduce the thermal hazards of polymer matrixes to some extent [30,31,32]. Graphene and its derivatives have ultra-high thermal stability and can play a flame-retardant role in their condensed phase. Their flame-retardancy mode of action is that they are layered two-dimensional materials, which can act as a barrier to inhibit heat transfer to the degrading polymer matrix [33,34,35]. However, there is competition between the barrier effect and the high thermal conductivity of rGO. Moreover, adding graphene or its derivatives alone often cannot meet the flame-retardancy requirements of polymers. They are often used in a way that enables a cooperative flame-retardant effect with other materials [36].

**Figure 2:**
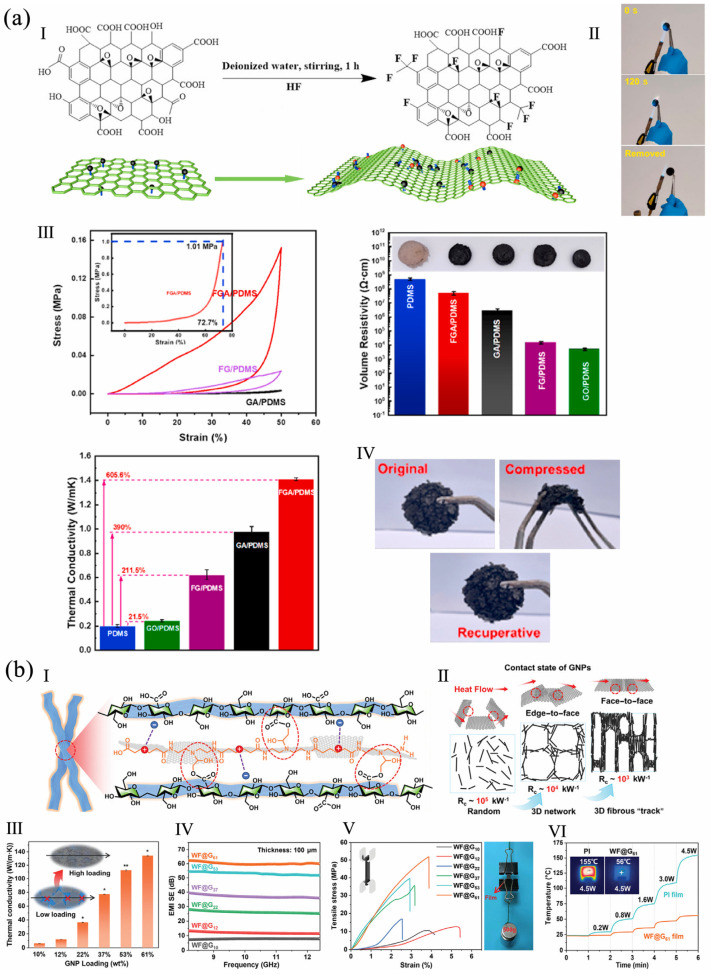
(**a**) FGA/PDMS: (**I**) preparation of FGA, (**II**) FGA/PDMS during flaming, (**III**) stress–strain cyclic curves, volume resistivity, thermal conductivity of the FGA/PDMS, (**IV**) FGA/PDMS before/after compressing, reproduced with permission from [38], Elsevier, 2022. (**b**) WF@G: (**I**) crosslinking network of WF@G, (**II**) contact states of GNPs and calculated thermal resistances, (**III**) thermal conductivity (* represents significant differences, * *p* < 0.05, ** *p* < 0.01), (**IV**) EMI SE of WF@G_n_ (_n_ represents the mass percentage of GNPs), (**V**) tensile curves, (**VI**) temperatures and thermal images of circuit with polyimide (PI) and WF@G as substrates, reproduced with permission from [39], Wiley, 2023.

A graphene/polydimethylsiloxane (PDMS) foam was prepared using a nickel-based template. The conductive foam with a hierarchically porous structure had a high stretchability < 160%, hydrophobicity with a contact angle > 126°, shock-absorbing ability > 300 kPa, and high flame-retardancy of only 6.5% weight loss up to 400 °C. The foam with graphene formed a compact and uniform char during combustion [37]. In another study, fluorinated GA/PDMS (FGA/PDMS) was prepared by freeze-drying and vacuum impregnation (Figure 2a). The obtained material was not damaged after burning at 1982 °C for 120 s. With 0.28 wt% of FGA, the material formed a thermal conduction network and a high thermal conductivity of 1.41 W m^−1^ K^−1^ was reached; the increment was 605.6% compared to pure PDMS. The material had high compressibility and a low electrical conductivity which was 180 times smaller than that of GA/PDMS [38].

Wood fibers (WFs) were coated with GNPs/polyamide epichlorohydrin (PAE) by electrostatic self-assembly and hot-pressing to form WF@GNP/PAE (WF@G). A low thermal contact resistance was achieved because of the close face-to-face contact between the GNP layers (Figure 2b). It had flame-retardancy with a limiting oxygen index (LOI) value of 65%, an ultra-high thermal conductivity of 134 W m^−1^ K^−1^, a mechanical strength of 50 MPa, an electromagnetic interference (EMI) shielding efficiency (SE) of 90 dB at 0.7 mm, and Joule heating performances [39]. In another study, a carboxymethyl cellulose (CMC)/rGO aerogel was prepared by in situ borate crosslinking and freeze-drying. The CMC/rGO aerogel was a nonflammable material with a LOI value of 30.4%. The compressive strength reached 128 kPa, five times that of CMC aerogel without rGO. It also had a good oil adsorption capacity of 90 g g^−1^ at 30 °C [40].

Coatings based on graphene and its derivates were used to enhance the overall performance of polyurethane (PU) foams. For instance, a PU foam was dip-coated with GO and silicone resin (SiR) solution and then cured. With 1 wt% of GO, the peak of heat release rate (PHRR) of the foam decreased by 65.84%, and the LOI increased to 29.8%. The foam self-extinguished and formed three layers after burning. The outer layer had porous nano-silica and char which suppressed heat and oxygen transfer. The expanded porous middle layer was a buffer zone for accommodating volatile flammable substances, as result of which the inside layer was protected [41]. A polydopamine (PDA)/rGO with micro-nano roughness was coated on a PU sponge skeleton via in situ polymerization, then the PDA/rGO coating was grafted with a silane coupling agent. The obtained coating was superhydrophobic with a water contact angle > 160 °. Its strong electrical conductivity persisted even after 1000 loading cycles. The coating also had high flame-retardancy because of the cooperative effect of the radical capture capacity of PDA and the barrier effect of rGO. The fire extinguished within 42 s without a melt droplet [42].

**Figure 3:**
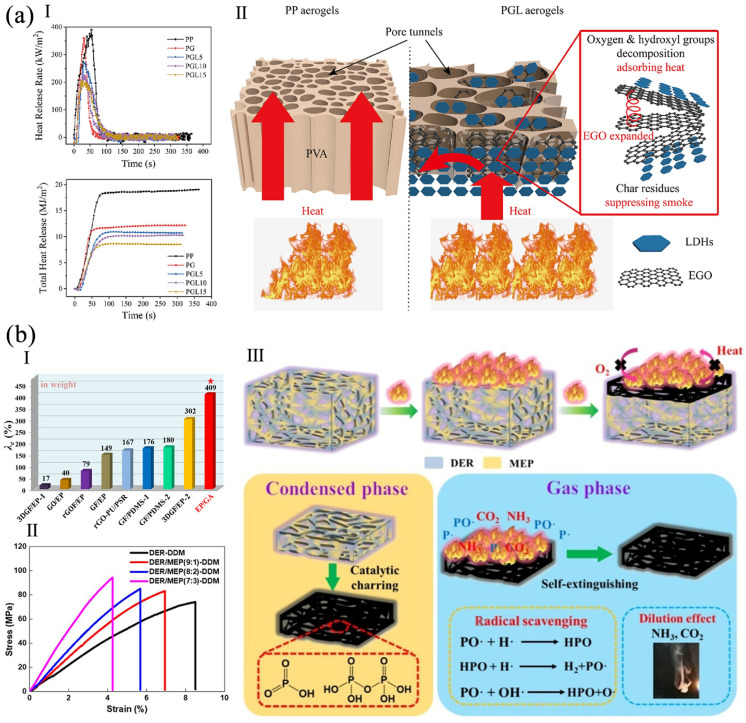
(**a**) PGL aerogels: (**I**) HRR and THR of polypropylene (PP), PVA/EGO (PG), PGL, (**II**) flame-retardancy mechanism reproduced with permission from [44], Elsevier, 2023. (**b**) EP/GA: (**I**) comparison of thermal conductivity of EP/GA (the red star), and three-dimensional graphene fillers (3DGF), graphene foam (GF), rGO-coated foam (rGOF), polysulfide rubbers (PSR) modified EP and PDMS with different filler content, (**II**), flexural stress–strain curves of EP/GA with different amount of diglycidyl ether of bisphenol A (DER), epoxy monomer (MEP), and 4,4′-diaminodiphenylmethane (DDM), (**III**) flame-retardancy mechanism of EP/GA reproduced from [45], American Chemical Society, 2021.

When the improvement in flame-retardancy using a single flame retardant does not meet the requirements, graphene and its derivatives are often used for a synergistic effect. For example, a polyvinyl alcohol (PVA)/expansible GO (EGO)/layered double hydroxides (LDH) aerogel, named PGL, was fabricated by freeze-drying and electrostatic adsorption. The PGL aerogel had high flame-retardancy due to the capacity to absorb heat, as well as the charring and barrier effect, of EGO and LDH (Figure 3a). It reached a V-0 rating in an Underwriters Laboratories 94 (UL-94) test [43] and a LOI of 31.0%. Its PHRR, total heat release (THR), and total smoke production (TSP) declined by 47.6%, 55.4%, and 54.3%, respectively. By creating hydrogen bonds, LDHs stabilized the pore structure and enhanced the compressive strength and modulus [44]. A thermal conductive vanillin-based epoxy resin (EP)/GA was fabricated. The aerogel had a LOI of 27.5% and reached a UL-94 V-0 rating. The THR and PHRR declined by 27% and 35%, respectively. This was ascribed to the char layer, which impeded oxygen and heat transfer, the quenching action of PO^•^ and PO_2_^•^ radicals, and the noncombustible gas dilution effect (Figure 3b). The thermal conductivity of the aerogel with 0.5 wt% of graphene was 0.592 W m^−1^ K^−1^. Its flexural strength and modulus increased by 24.0% and 115%, because of the rigid epoxy monomer and hydrogen bonding connection [45]. An ammonium polyphosphate (APP)/GA was grown in situ on polyethylene terephthalate (PET) by hydrothermal reduction. With 26 wt% of APP/graphene, the LOI of the aerogel was 34.1%, and the PHRR, THR, and TSP were reduced. The char residual increased to 91.5% after combustion. The APP/graphene formed a dense protective layer on the surface of PET, improving its flame-retardancy [46].

By optimizing the well-designed structure of blends, and combining graphene with other flame retardants, materials with excellent flame-retardancy and improved mechanical properties were obtained. However, methods to accurately control the amount of graphene, improve the adhesion between graphene coatings and polymer matrixes, increase the multifunctionality of the blends, and reduce costs are worthy of further research.

## 3. Expanded Applications of Porous Flame-Retardant Materials Containing Graphene

### 3.1. Firefighter Clothing

The vital equipment that helps keep firefighters safe when fighting fires is firefighting protection clothing. Firefighting apparel must delay the ambient heat flow from reaching human skin [47]. However, it becomes challenging to evacuate safely and on time if high-temperature flames destroy firefighting clothing. Hence, developing a real-time flame warning material that provides an early warning before the thermal decomposition of firefighting clothing is needed [48]. Moreover, the classical multi-layer fire-protective clothing is heavy and makes movement inflexible. It is necessary to design a new type of fabric with low density, high durability, and good flame retardant and thermal insulation properties to provide better safety for firefighters [47].

Aramid fiber is the traditional material of firefighting protection clothing, as it is capable of dealing with temperatures of 400 °C, but it cannot provide an early warning [48]. Phase-change materials (PCMs) have considerable promise as temperature-regulating materials, because they are active heat storage–release materials that are capable of absorbing and releasing energy by phase change [49]. They are also known to reduce heat and give early warning. Moreover, the phase-change temperatures of organic PCMs are similar to human body temperature, which makes them appropriate as firefighting textiles [47]. Foam or aerogel based on graphene and its derivatives can be used as the framework to encapsulate PCMs, and can also provide an effective heat transfer network [50]. 

### 3.2. Fire Alarm Sensor

A prompt and accurate fire alarm is essential for effective fire rescue during a fire hazard [51]. The most popular indoor fire detectors are infrared and smoke detectors. Nevertheless, their sensitivities are easily affected by high-temperature and wet environments. They have low penetration in water vapor, smoke, and other gas molecules, which can result in false alerts [52]. Moreover, their reaction time usually exceeds 100 s because of the high recognition threshold, and the alert can only trigger when the fire produces a lot of smoke. Thus, developing a new fire alarm that operates steadily and efficiently is crucial [51].

Because of its distinct electronic structure, graphene has great electrical conductivity. GO is an electrical insulator since it is altered chemically [53]. During combustion, GO is easily reduced. Its oxygen-containing groups will be eliminated and it will be reduced to rGO, restoring its conductive structure [52]. This transformation can be used in fire warning systems to provide quick identification and early alert during the pre-combustion period [54]. 

Research has been focused on the temperature-resistance property of GO [55,56,57,58]. Various fire-detection sensors containing GO, such as thermosensitive papers, fire-resistant foams, and large-area coating textiles, have been developed to provide early fire alarms [48]. However, it is still difficult to produce self-sustaining, highly sensitive, enduring, and versatile fire warning devices. Further research and improvement in this area is being conducted by researchers. 

### 3.3. Flexible Electronic Skin

Wearable piezoresistive sensors with flexibility and low density can simulate natural skin to enable the real-time and quick detection of physical stimuli. They have the potential to be used in a variety of applications, including humanoid robotics, prosthetics, wearable health-monitoring systems, soft robotics, etc. [2,59]. Piezoresistive devices are often energy saving and easy to process, and have a quick reaction and high sensitivity. The piezoresistive process is a reversible evolution in a conductive network, which refers to the response of resistance caused by deformation as a result of outside stimuli [60,61].

3D porous conductive graphene sponges, foams, and aerogels are good options for making piezoresistive sensors due to their feasible preparation, being lightweight and low cost, excellent elasticity, and conductivity. However, carbon nanomaterials tend to stack and aggregate, and their network structures are difficult to regulate, so the endurance and elasticity of as-prepared piezoresistive sensors are weakened [60]. Moreover, 3D porous structures are unable to withstand high pressure due to their low density, so the as-prepared piezoresistive sensors are not sensitive in high-pressure areas [2]. Moreover, their being portable and wearable, and their tolerance of high-temperatures, also needs to considered [60].

### 3.4. Solar Energy Storage

Nowadays, the shortage of fossil fuels and the energy crisis have grown to be significant worries for human civilization and the global economy. Using sustainable energy can help to address this [62]. Solar energy is a widely accessible and endless source of renewable energy. However, the practical use of solar energy is restricted by low solar–thermal conversion and mismatching in time and space [63].

PCMs can reduce emissions and save energy by keeping or releasing latent heat during a phase transition. They can also improve the imbalance of supply and demand of energy [64,65]. Through the energy transfer of solar–thermal, electro–thermal, and solar–thermal–electricity, they can significantly boost the usage of energy [66,67,68]. Thus, PCMs offer prospective applications in building warming materials, solar energy storage, energy-saving air conditioning, etc. They improve the utilization of solar energy without environmental constraints [62,63].

Currently, the three most popular embedding methods for organic PCMs are solvent casting, microcapsule encapsulation, and the porous media adsorption method [64,69]. The porous media adsorption method can maintain form stability and achieve steady thermal energy conversion. Graphite foam, expanded graphite, and other polymer blends are the common porous media [62]. However, the carbon materials often agglomerate and precipitate in PCMs, which reduces the energy storage ability and thermal conductivity of polymer matrixes [66].

Researchers have used GA to adsorb PCMs, which solved the dispersion problem of graphene and provided an effective thermal transfer network and flame-retardancy [64,70]. PCMs are embedded in the pores of GA with a high latent heat of phase transition and no leakage [62,71,72]. The graphene thermal network can enhance the thermal conductivity, offer sites for nucleation, and allow for the storage of solar energy due to the photothermal conversion capacity of GA [63].

### 3.5. Energy-Efficient Building

Thermally insulating materials are becoming a research hotpot in construction due to the increasing need for energy-saving buildings [18,53,73]. This serves as an obstacle to block heat transmission caused by temperature gradients. There are many insulating materials for building, including expanded polystyrene, PU, polypropylene, etc. However, these materials have poor thermal-oxidative stability, are easily ignited, exhibit structural collapse, and induce fire accidents [42,51]. The toxic gases released during their combustion are also deadly and detrimental to escape [52]. Currently, being lightweight, exhibiting low thermal conductivity, flame resistance, and being mechanically resilient or flexible are the basic evaluation standard for external wall insulation materials [74,75,76,77].

Porous materials were employed to regulate the energy efficiency of buildings. Aerogels are an excellent option for thermal isolation because of their 3D network structure, ultralow density, high specific surface area, and ultralow heat conductivity [52]. Many researchers are devoted to incorporating graphene and its derivates into porous polymer foam and aerogel to develop nontoxic, flame-retardant, and thermal insulating porous material for energy-efficient buildings [78].

### 3.6. Stealth Material

Human health and national security are at risk from electromagnetic (EM) radiation [79,80]. Strict standards for EM shielding and absorbing materials have been established due to the quick growth of flexible devices and stealth materials [1,81]. For example, a high mechanical strength of EM wave-absorption materials is required to withstand the impact of air waves when they are coated on the surfaces of airplanes and satellites to evade radar detection. Flame-retardancy is also needed due to the fast rise in surface temperature during flying. Developing absorbing materials that are thermally insulating is also crucial for maintaining their absorption ability at high temperatures. Moreover, with the miniaturization, integration, and intelligence of electronic devices, EM-shielding materials need to be flexible and lightweight and have thermal stability [20].

It is ideal to develop advanced EM absorption materials that are lightweight and have a broad bandwidth, high absorption intensity, and thin matching thickness [82,83]. In the past few decades, significant development has been made in the preparation and application of EM-absorbing materials. However, realizing the remarkable overall performance remains challenging [84].

Graphene and its derivatives are widely used in EM shielding and absorption because of their low density, high stability, high electrical conductivities, mechanical strength, high specific surface area, and good ferromagnetism, as well as exhibiting the quantum Hall effect [1,20,82,83]. Furthermore, they have hydrophobicity, thermal isolation, and flame-retardancy [79]. However, individual fillers were unable to meet the demands of sophisticated materials [85]. Hence, 3D porous materials containing graphene and its derivatives were combined with others to a realize high EM absorption capacity and broadband [79,86].

### 3.7. Separation

Carbon dioxide, organic pollutants, and oil spills all require adsorption and separation treatment. Because of the global use of fossil fuels, carbon dioxide is a key reason for the greenhouse effect [87]. Organic pollutants and oil spills lead to damages in ecology and finance, fire risk, or even explosions [88,89]. Because adsorption is low-cost and can be used in high-temperature and -pressure environments, it is an effective way to remove organics, oil spills, and CO_2_.

Conventional absorbents have some drawbacks, such as an insufficient absorption capacity and a poor environmental resistance and cycle performance. Researchers have constructed rough structures on the surfaces of sponges or foams to achieve easy processing, tunable pores, high hydrophobicity, quick absorption, and efficient recyclability [90].

Porous graphene-based materials are popular adsorbents because they have the potential to meet the above requirements [26,91]. For example, GAs are ideal adsorbents for dyes, oils, heavy metals, and pollutant gases [87]. They function as an ideal filter and have a remarkable oil absorption capacity, about hundreds of times their own weight [90].

In summary, graphene-based porous materials with multiple energy-conversion properties are expected to be used in firefighter clothing, fire alarm sensors, flexible electronic skin, and solar energy storage. Graphene-based porous materials that exhibit thermal insulation, electromagnetic shielding, and a high adsorption capacity have the potential to be used in energy-efficient buildings, stealth material, and separation, respectively. However, the integration of high mechanical properties and multifunctional materials remains a challenge in practical use. It is necessary to investigate the key factors that govern porous networks, microstructures, and mechanisms for functional performance in future studies, and to realize the required multifunctionality.

## 4. Improved Properties of Porous Flame-Retardant Materials Containing Graphene

### 4.1. Multiple Energy Conversion Properties

By utilizing the multiple energy conversion properties, porous flame-retardant materials containing graphene are potential candidates in fields such as firefighter clothing, fire alarm sensors, flexible electronic skin, and solar energy storage [92]. Researchers are committed to improving the flame retardant, photothermal, and piezoelectric properties of graphene-based porous materials to expand their high-end application range.

**Figure 4:**
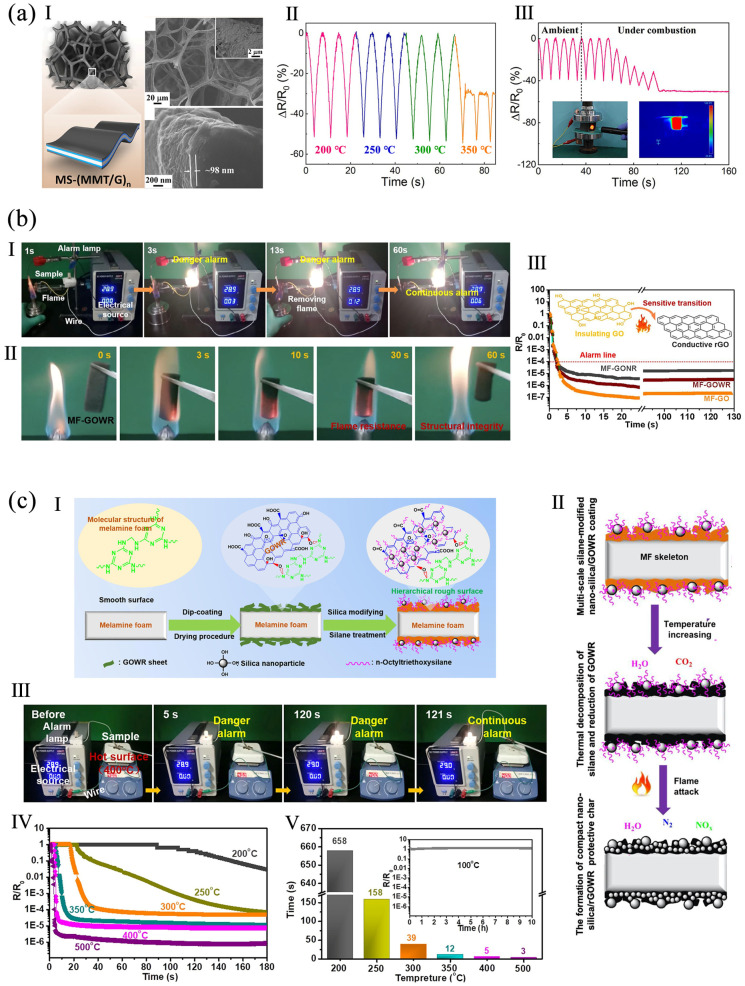
(**a**) MS-(MMT/G): (**I**) structure and morphology, (**II**) relative resistance change in the sensor, (**III**) durability test, optical and infrared images of the sensor under combustion reproduced from [60], American Chemical Society, 2021. (**b**) MF-GO sponge: (**I**) flame detection and alarm device based on MF-GO, (**II**) burning process of MF-GO wide-ribbon, (**III**) electrical resistance changes in MF-GO reproduced with permission from [51], Elsevier, 2021. (**c**) Silica/GOWR@MF: (**I**) the fabricating process, (**II**) during high temperature and flame attack, (**III**) Photographs of silica/GOWR@MF sponge for detecting the hot surface of 400 °C, showing a rapid flame alarm release at 5 s and continuous alarm response, (**IV**) electrical conductivity changes, and (**V**) response time reproduced with permission from [88], Elsevier, 2021.

Montmorillonite (MMT) and graphene were alternatively coated on melamine sponge (MS) scaffold to form a MS-MMT/G blend (Figure 4a). The blend showed high thermal stability at 250 °C for 7 days, showing stable and real-time human-motion detection under combustion for 20 s. It had a deformation-tolerant conductivity at 80% strain. It also acted as a piezoresistive device, exhibiting a high gauge factor of 2.3 at 60–80% strain and a durability of 10,000 cycles at 60% strain without impairing its piezoresistive property [60]. MF sponges were coated with GO derivatives by a dip-coating approach. The GO derivatives improved the sponges’ hydrophobicity (water contact angle of 130°) and compression property. The sponges also showed high flame-retardancy, maintained their original structure during combustion, and were used in a fire alarm sensor (Figure 4b). The MF-GO nanoribbon sensor had a quick response time (6.3 s) compared to MF-GO wide-ribbon (MF-GOWR) (8.4 s) and MF-GO sheet (11.1 s) sensors, because the highly interconnected network of GO nanoribbon had a shorter thermal reduction process [51]. In another study, a silica/GOWR-coated MF sponge (silica/GOWR@MF) was prepared. The GO was dip-coated on a MF skeleton, then surface-modified by nano-silica (Figure 4c). The silane generated silica during combustion and formed a snowflake-like layer on the MF. The sponge had a fast response of 3 s and a stable warning signal (39 s for 300 °C) in fire. It also had super-hydrophobicity/super-oleophilicity, exhibiting the ability to absorb heavy and floating oils from water, and could continuously separate water and oil [88].

**Figure 5:**
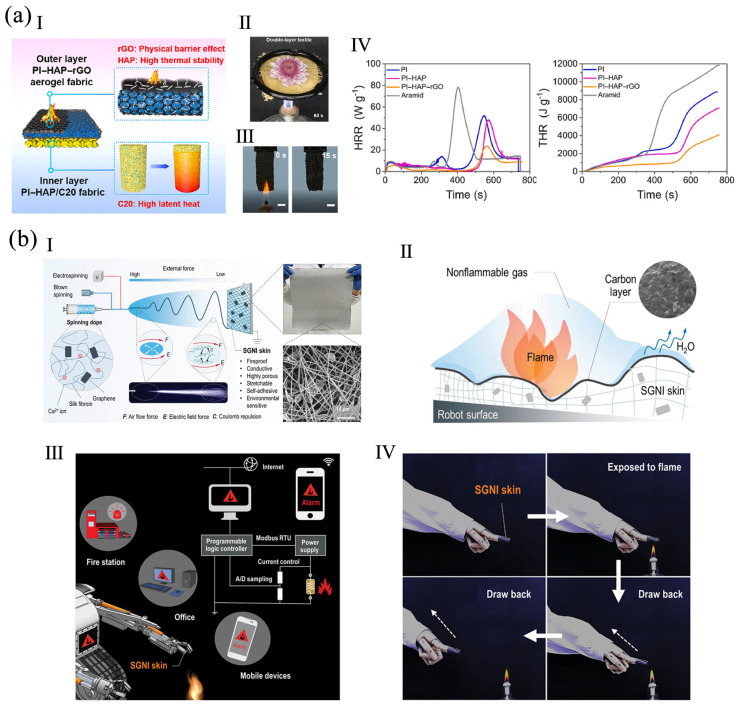
(**a**) A double-layer textile with PI-HAP/C20 and PI-HAP-rGO aerogel as inner and outer layers: (**I**) the function of the textile, (**II**) a photo of the textile indicating thermal insulation, (**III**) PI-HAP-rGO after ignition, (**IV**) HRR and THR of PI-HAP-rGO reproduced with permission from [47], Elsevier, 2023. (**b**) A SGNI skin: (**I**) the fabrication method, (**II**) flame-retardant mechanism, (**III**) schematic of a fire alarm system, (**IV**) actions triggered by high temperature on SGNI skin reproduced with permission from [93], Wiley, 2021.

A sodium alginate (SA)/hydroxyapatite (HAP)/GO aerogel was prepared. Compared with SA aerogel, the THR and PHRR of the SA/HAP/GO aerogel were reduced by 40.9% and 42.9%, respectively. The catalytic charring role of HAP and barrier effect of GO slowed down the mass and heat delivery. Aerogels are electrically insulated at room temperature, but conduct electricity when exposed to flame, because GO restores conductivity by thermal reduction. The SA/HAP/GO aerogel responded extremely quickly, within 1.5 s, and, alarmingly, for more than 60 s. The aerogel can operate at a maximum temperature of 700–800 °C [53]. In another study, a textile comprising phase-changeable polyimide-hydroxyapatite/C20 (PI-HAP/C20) as its inner layer and PI-HAP-rGO aerogel as its outer layer was fabricated (Figure 5a). The outer layer had a high flame-retardancy, with a LOI of 47.5%, and its PHRR was decreased by 70.0% compared to aramid fabric. The inner layer had a high C20 loading of 93 wt%, endowing high melting enthalpy which suppressed temperature rise by the thermal buffering effect. Therefore, the obtained textile can prolong the pain threshold of human skin (280 s) [47].

A silk/graphene nanoionotronic (SGNI) skin was created using an electro-blown spinning technique (Figure 5b), which was more efficient than electrospinning. The porous SGNI skin was extremely stretchy, mechanically strong, conductive, and sensitive to temperature and humidity. Due to the inclusion of chelated Ca^2+^ and silk, it had humidity-induced self-adhesiveness. The SGNI skin with graphene had good flame-retardancy, and it was assembled as a fire warning, achieving real-time alert within 2 s for phones, the cloud, and a central control system [93]. In another study, a cellulose nanofiber (CNF)/APP/GO aerogel was prepared via freeze-drying. It had outstanding flame-retardancy and thermal isolation. When GO and APP were incorporated, the THR and PHRR values were reduced by 40.7% and 78.6%, respectively. A fire-warning detector based on this aerogel was designed. Because of the transition in the conductivity state of GO under flame exposure, it had an alarm within 2.6 s and emitted a long-lasting alarm signal [52].

Graphene-based aerogel has a remarkable adsorption ability, thermal conductivity, and good mechanical characteristics. Research has integrated graphene-based aerogel into PCMs to improve their heat conduction capability and their chemical and thermal stability, and to prevent their leakage [94,95].

GA was prepared and compounded with paraffin wax (PW) by high-temperature annealing and vacuum-assisted impregnation. The as-prepared phase-change material had a PW loading of 98%, an improvement of 44.44% in thermal conductivity, and a reduction of 41.3% in PHRR. Moreover, the material had photothermal conversion capability, and its temperature increased by 43 °C after 4 min of light exposure [64]. Graphene/boron nitride (GB) aerogel was fabricated via self-assembly during calcination and incorporated with PW to obtain a PW/GB phase-change material. The initial thermal decomposition temperature (T_5%_) and the maximum thermal decomposition temperature (T_max_) of the PW/GB material reached 196.0 °C and 286.6 °C because of the thermal stability and barrier effect of GB aerogel. The dual thermal conductivity networks of GB provided a high latent efficiency of 96.7% and a low leakage rate of 3.0% compared to PW-GB. The thermal conductivity reached 0.4108 W m^−1^ K^−1^, 81.5% higher than that of pure PW because of the highly thermally conductive boron nitride attachment [63]. In another study, silver nanoparticles (AgNPs)/GA was prepared by hydrothermal reaction and thermal annealing, and then vacuum impregnation was used to encapsulate PW. The 3D porous graphene assisted in the dispersion of AgNPs, and provided efficient heat transmission routes, thermal storage, and solar–thermal and electro–thermal conversion abilities. In comparison to pure PW, the modified PW exhibited a decrease of 30.2% in PHRR, a latent heat retention of 97%, an improvement in thermal conductivity of 39.35%, and a solar–thermal and electro–thermal conversion efficiency of 92.62% and 95.19%, respectively [66].

A graphene-modified PVA (GP) aerogel was fabricated for encapsulating polyethylene glycol (PEG). After heating at 70 °C for 1 h, the leakage rate was only 1.13%, which is lower than that of many other reported aerogel-based PCMs. Compared to pure PEG, the as-prepared aerogel had decreases of 37.5% and 25% in PHRR and THR, because of the barrier effect of graphene. The thermal conductivity was about 1.6 times that of PEG. The solar–thermal conversion increased because of the light captor effect of graphene, and the aerogel was 50 °C after 80 s of solar irradiation [50].

Graphene was coated on APP/EP by laser scribing. In the laser scribing procedure, the C-O, C=O, P-O, and N-C bonds were broken, while the remaining C atoms recombined to construct a porous hierarchical graphene layer. The TTI of the obtained material increased by 36 s, the LOI was raised to 37%, and the PHRR, THR, and TSP were reduced by 71.1%, 75.2%, 74.1%, and 40.7%, respectively. A supercapacitor with the as-prepared APP/EP/graphene electrode had a mass-specific supercapacitance of 245.6 F g^−1^ [96]. In another study, multiple-graphene-shell-wrapped Si-based material with a spherical structure was synthesized by spray-drying, and was fabricated as a rGO/Si/GA anode for Li-ion batteries. The rGO facilitated the electron movement from Si to electrolytes, and inhibited the continuous creation of an unstable solid–electrolyte interface layer. The anode had a strong cycle stability, rate capability, and initial discharge capacity [97].

In summary, porous graphene-based materials have fire warning potential because they can be reduced and transform from an insulating state to a conductive state when exposed to flame. They can also be used to create advanced PCMs. By integrating their multiple energy conversion properties like their piezoelectric effect, and solar–thermal and electro–thermal conversion, the application scope of porous graphene-based materials has been further expanded.

### 4.2. Thermal Insulation Property

Due to the sharp rise in energy consumption, energy-efficient buildings require thermally insulating materials. To address this, a balance of high strength, good flame-retardancy, and poor thermal conductivity is required. Many researchers are devoted to incorporating graphene and its derivates into porous polymer foam and aerogel to develop nontoxic, flame-retardant, and thermally insulating porous materials for energy-efficient building [52,78]. For instance, PU foam is easily lit and burns through in a matter of seconds, along with continuous dripping and rapid flame self-propagation. Additionally, it releases a large amount of heat and produces poisonous fumes and molten products [41]. Graphene and its derivates were used to improve the overall performance of PU foam. However, graphene, as a carbon material, can significantly increase the thermal conductivity of the polymer. There is competition between the barrier effect and the high thermal conductivity of graphene, and solving this issue is crucial.

Phosphorus-containing GO/PU foam was fabricated via vacuum impregnation. Compared to pure polyurethane foam (PUF), the PHRR, THR, and maximum of specific optical density of smoke of the foam were reduced by 55.3%, 26.5%, and 45.18%, respectively, and the LOI increased by 57.1%. The foam retained its thermal insulation property with a thermal conductivity of 0.030 W m^−1^ K^−1^. The sound-absorption effect was improved, and the average sound-absorption coefficient was 245.45% greater than that of PUF [98]. In another study, A PU/GNPs/aluminum open-cell foam was fabricated. The foam had a low thermal conductivity of 0.038 W m^−1^ K^−1^ and strong sound absorption, particularly at mid-to-high frequencies [15]. A PU/graphite aerogel was prepared to enhance the thermal insulation and flame-retardancy of PU. A 20% reduction in thermal conductivity was observed. The aerogel exhibited a 10% reduction in its surface peak temperature [99].

**Figure 6:**
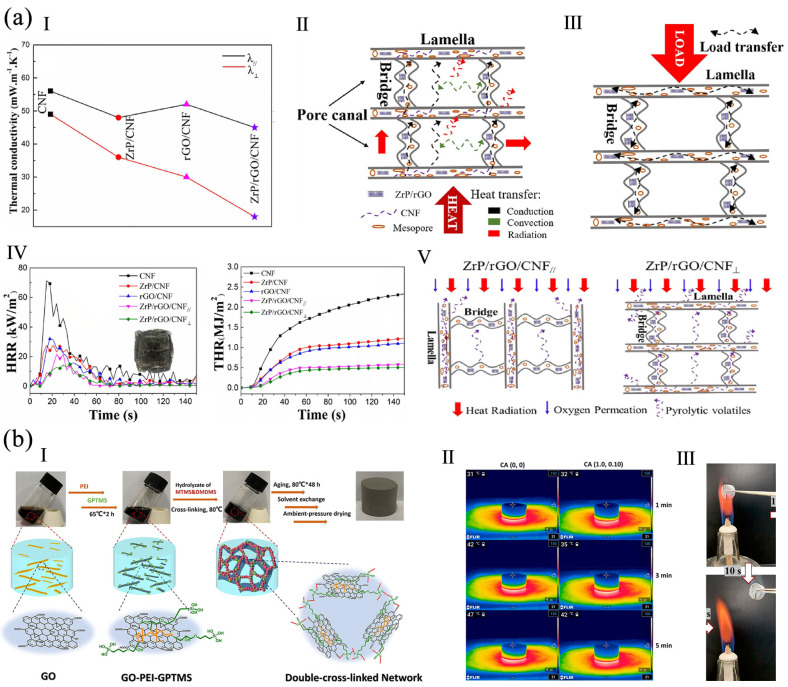
(**a**) ZrP/rGO/CNF aerogels: (**I**) thermal conductivities of the aerogels, (**II**) factors affecting thermal conductivity, (**III**) the load transfer in the aerogel, (**IV**) HRR and THR of ZrP/rGO/CNF, (**V**) combustion process in parallel and vertical directions reproduced with permission from [75], Elsevier, 2020. (**b**) A GO/silica-based aerogel: (**I**) the fabrication method, (**II**) infrared thermography images of PEI and GO-PEI-GPTMS during heating, (**III**) GO-PEI-GPTMS aerogel during combustion, reproduced from [14], American Chemical Society, 2020.

A CNF/GO/MMT aerogel was prepared by vacuum freeze-drying. The aerogel was pressure-proof and had a thermal conductivity of 39 mW m^−1^ K^−1^. It had flame-retardancy because GO/MMT inhibited the transmission of heat and oxygen. A fire warning system based on the aerogel was fabricated. When it was attacked by fire, the GO was reduced to conductive rGO, and the fire-alarm was triggered in 1.9 s and persisted for 137 s [54]. In another study, hierarchical zirconium phosphate/graphene (ZrP/rGO) nanosheets were prepared by a spatial confinement method, then a ZrP/rGO/CNF aerogel was assembled via freeze-casing. The aerogel had a low thermal conductivity, a high LOI of 33.5%, and a low PHRR of 14.1 kW m^−2^ (Figure 6a). The char residue was also improved. The thermal stability was enhanced and the release of pyrolytic volatiles was reduced [75].

GO/silica-based aerogel was prepared by the sol–gel method. GO was modified by 3-glycidyloxypropyltrimethoxysilane (GPTMS) and polyethylenimine (PEI). After a cohydrolysis and condensation of trimethoxymethylsilane (MTMS) and dimethoxydimethylsilane (DMDMS) in the presence of GO, an aerogel with a double-crosslinked network was obtained (Figure 6b). With 0.5 wt% of GO, the Young's modulus was five times that of a pure polysiloxane aerogel, the maximum degradation temperature was raised 90 °C, and the thermal conductivity was 0.049 W m^−1^ K^−1^. The aerogel also showed stable piezo-resistive behavior [14]. rGO-doped silica aerogel (SA/rGO) was prepared. The rGO enhanced the flame-retardancy of SA aerogel while maintaining its thermal insulation with a low thermal conductivity. The onset and peak decomposition temperatures increased by 118 °C and 94 °C, and the THR decreased by 25.4%, compared to pure SA aerogel. Competition between the barrier effect and the high thermal conductivity of rGO existed [30]. The APP, SA aerogel, and expandable graphite (EG) were used to functionalize rigid PU to form EG/APP/SA/rigid PUF (RPUF) foams. The foam achieved a V-0 UL-94 rating, the char residual was increased by 60.9% at 700 °C, and the THR and TSP were reduced by 9.2% and 17.5%. With 1 wt% of SA, the compressive strength of the foam was increased, and the thermal conductivity was reduced to 19.8 mW m^−1^ K^−1^ [74].

**Figure 7:**
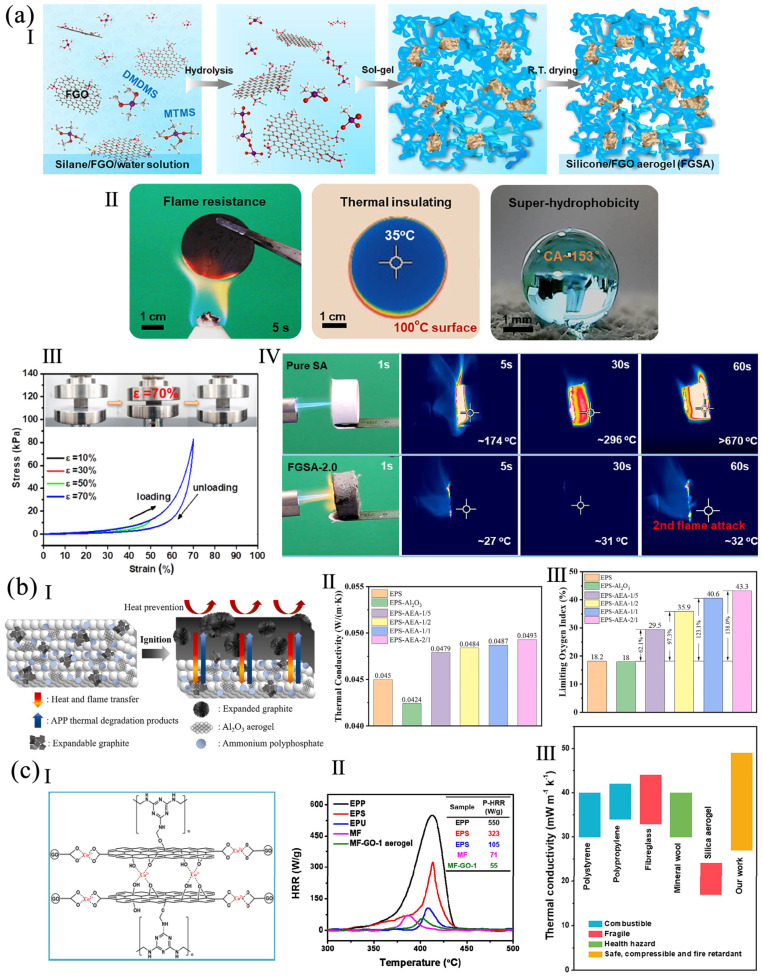
(**a**) Silicone/FGO aerogel: (**I**) the fabricating process, (**II**) the images display the flame-retardancy, thermal insulation, and super-hydrophobicity, (**III**) compressive stress-strain curves and their recovery images at different compressive strain values (ε), (**IV**) fire attack and thermal insulating images reproduced with permission from [78], Elsevier, 2022. (**b**) EPS/Al_2_O_3_/EP/EG/APP: (**I**) the flame-retardant mechanism, (**II**) the thermal conductivity, and (**III**) LOI reproduced with permission from [101], Elsevier, 2023. (**c**) MF-GO aerogel: (**I**) its structure, (**II**) HRR, (**III**) thermal conductivity, reproduced with permission from [102], Elsevier, 2021.

rGO/siliconboron carbonitride (SiBCN) aerogels were fabricated by solvothermal treatment, freeze-casting, and pyrolysis. Because of their hierarchical structure, the aerogels had a low thermal conductivity of 0.057 W m^−1^ K^−1^, good thermal stability at 1200 °C in argon, melting resistance, and short oxidation resistance [100]. A silicone aerogel was combined with γ-aminopropyltriethoxysilane-modified GO (FGO) by a sol–gel and drying method (Figure 7a). The APTES promotes the dispersion of GO. Because of the large specific surface area of FGO and strip-like co-crosslinking networks, the aerogels had flame-retardancy (rapid self-extinguishing), super-hydrophobicity, compressive flexibility, a strong thermal insulation performance, and oil/water separation capacity, as well as being recyclable [78]. 

An expandable polystyrene (EPS)/Al_2_O_3_/epoxy resin/EG/APP was prepared. The blend has a LOI of 43.3% (Figure 7b), and ranked V-0 in the UL-94 test. The THR, TSP, CO, and CO_2_ releases decreased by 94.1%, 97.7%, 85.9%, and 90.3%, respectively, compared with EPS. EG played a physical barrier effect, and APP produced ammonium and phosphoric acid and prevented oxygen transfer. Al_2_O_3_ reduced the thermal conductivity and improved the mechanical property. The blend had a compressive and tensile strength of 505 kPa and 283 kPa, and a low thermal conductivity of 0.0478 W m^−1^ K^−1^ [101]. A MF-based aerogel was prepared by using GO as a template skeleton and Co^2+^ as and acid catalyst and assembly-driven agent. Owing to its distinct hierarchical metal-MF and GO crosslinking network, the aerogel had high compressibility, with a compression strain of 80% and a high cycling stability. It also had a strong thermal insulation property, with a thermal conductivity of 27 mW m^−1^ K^−1^, and a lower PHRR of 55 W g^−1^ (Figure 7c) [102].

The PI/LDH-GO aerogel was fabricated via freeze-drying and thermal imidization. GO improved the LDH dispersion in PI and enhanced the mechanical property of the aerogel. The pore size of the aerogel decreased from 20 μm to 5 μm because of increased crosslinking sites generated by GO and PI chains. This resulted in low density, a low thermal conductivity of 36 mW m^−1^ K^−1^, and a high compressive modulus. It reached a nonflammable level with a reduced PHRR of 25.8 kW m^−2^, and the LOI was 43% [18]. Poly(m-phenylene isophthalamide) (PMIA) was used to prepare a graphene-based aerogel via freeze-drying. The PMIA appeared as micro particles that filled in the pores of the aerogel, hindering the self-assembly of the graphene. With the increase in PMIA content, the aerogel became denser and harder, with smaller pores and thinner graphene walls, so its compressive strength increased. It could bear 2000 times its own weight without deformation, and had a low thermal conductivity of 0.045 W m^−1^ K^−1^ [103].

### 4.3. Electromagnetic Shielding Property

**Figure 8:**
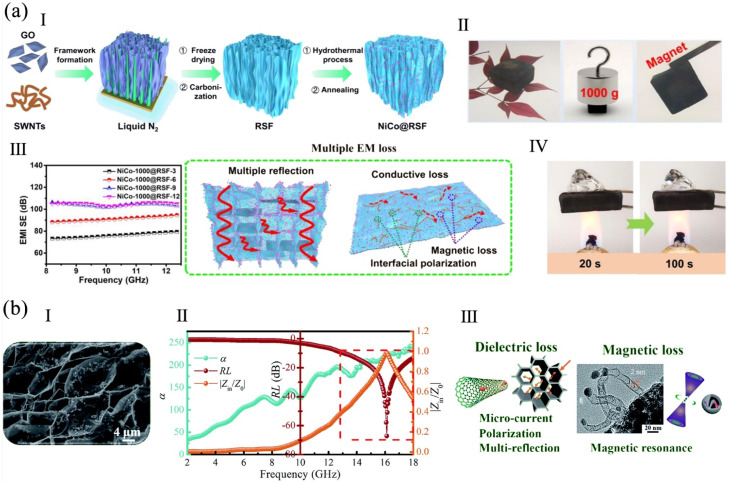
(**a**) NiCo@RSF: (**I**) the synthesis process, (**II**) the images of magnetic and ultralight NiCo@RSF with compression resistance, (**III**) the EMI SE and shielding mechanism, (**IV**) the burning images of NiCo@RSF, reproduced with permission from [86], Elsevier, 2023. (**b**) Co/CNTs/EG foam: (**I**) a SEM image, (**II**) the frequency-dependent attenuation constant (α), reflection loss (RL), and |Z_in_/Z_0_| values, Z_in_ and Z_0_ are the input impedance and free space impedance, (**III**) the EMI shielding mechanism, reproduced with permission from [84], Royal Society of Chemistry, 2021.

An oriented foam composed of a single-walled conductive skeleton (RSF) fabricated using CNTs and rGO was prepared, then magnetic NiCo nanoparticles were added to form a NiCo@RSF foam (Figure 8a). The EMI SE and specific SE (SSE/t) of the foam reached 105 dB and 18,711 dB cm^2^ g^−1^ at the X-band frequency. The foam also had heat insulation and flame-retardancy [86]. A sea urchin-like Co/CNTs/expanded graphite (EG) foam was fabricated. Due to the cooperative effect of the conduction loss attributed to electron transmission in the conductive CNTs/EG network, the magnetic loss from the Co, the dielectric loss from dipole/interfacial polarization, and multiple reflections of the porous framework (Figure 8b), the foam, at 1.4 mm, had a high reflection loss of 67.2 dB and a broad absorption bandwidth of 5.1 GHz. Moreover, the foam exhibited thermal insulation, flame retardant, and infrared shielding properties [84]. A honeycomb-like aerogel with inserted Co@C was prepared by freeze-casting and carbonization. The aerogel had robust mechanical performance with a longitudinal compression modulus of 1411 kPa at 80% strain. Due to good electrical conductivity, a large number of interfaces and dipoles, and a special organized porous structure, the aerogel at 1.5 mm had an EM absorption of 45 dB and an effective absorption bandwidth (EAB) of 4.02 GHz [83].

A sugarcane/rGO hybrid foam was prepared via vacuum impregnation and thermal annealing. With 17 wt% of rGO, the EMI SE, specific SE, and compressive strength of the foam were 53 dB, 3830 dB cm^2^ g^−1^, and 1.33 MPa, which were 36%, 13%, and 6% higher than those of annealed sugarcane because of its unique porous structure and abundant interfaces. It also had flame-retardancy, thermal stability, and a low thermal conductivity [104]. Flexible graphene/aramid nanofiber (ANF) carbonizing films were prepared via blade-coating and carbonization. The electron and phonon transfer routs were increased by the films’ highly oriented GNPs. The films reached a high thermal conductivity of 75.25 W m^−1^ K^−1^, 5700% higher than pure ANF film. With 5 wt% of graphene, the film, with a nacre-like structure at a 1.5 mm, had a minimum reflection loss (RL_min_) of −56.07 dB and a maximum EAB of 5.28 GHz [20].

**Figure 9:**
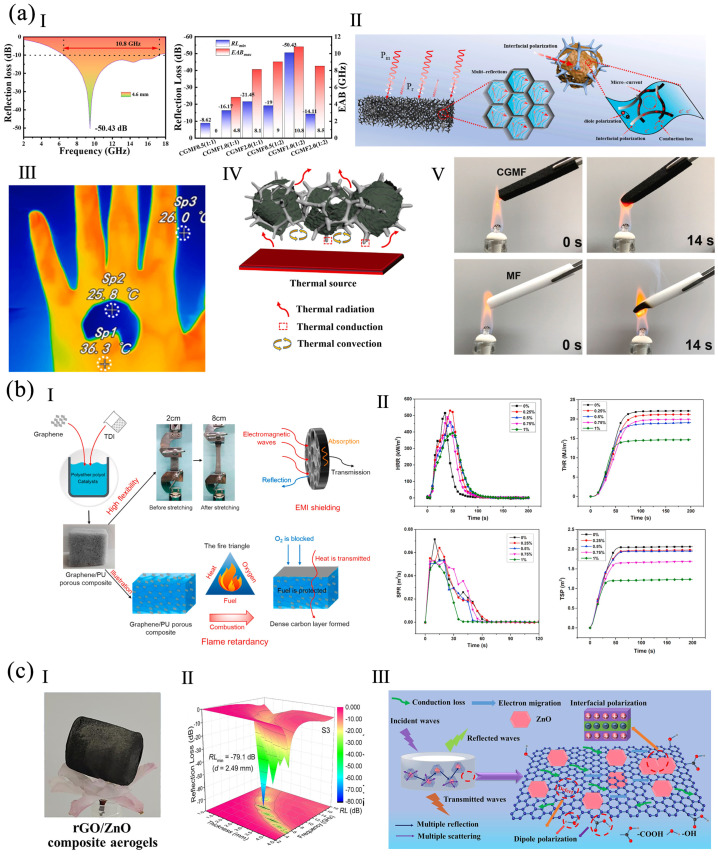
(**a**) CGMF: (**I**) RL curve, RL_min_, and EAB, (**II**) EM wave dissipation (P_in_ is incident wave, P_r_ is reflected wave), (**III**) a thermal infrared image for CGMF placed on a hand (the Sp1, Sp2 and Sp3 were three different area selected for testing), (**IV**) heat transfer mechanism, (**V**) CGMF and MF during combustion, reproduced with permission from [79], Elsevier, 2022. (**b**) A graphene/PU material: (**I**) EMI shielding and flame-retardant mechanism, (**II**) the HRR, THR, SPR, and TSP curves, reproduced with permission from [1], Elsevier, 2020. (**c**) rGO/ZnO aerogel: (**I**) appearance, (**II**) RL curves and contour map, (**III**) EM wave absorption mechanism, reproduced with permission from [82], Elsevier, 2022.

A multi-walled carbon nanotubes (MWCNT)/rGO-modified MF sponge was prepared. The MWCNT/rGO was attached on the 3D MF skeleton to construct a tri-continuous conductive network (CGMF). Broadband EMA properties were achieved by the cooperative effect of the EMW dissipation of the MWCNT/rGO network and good impedance matching from the 3D porous structure. (Figure 9a). The sponge, at a thickness of 4.6 mm, had an EABmax of 10.8 GHz and a RL_min_ of −50.43 dB. It also had good mechanical stability, hydrophobicity, heat insulation, and flame-retardancy [79]. A graphene/PU material with a polygonal open cell structure was prepared by the one-pot method (Figure 9b). The near- and mid-infrared transmittance of the obtained material was about 0%, and its low-frequency band EMI SE was 30 dB. The graphene enhanced the flexibility and flame-retardancy of the PU, with a 300% elongation at break and a 23.7% reduction of PHRR [1].

A rGO/ZnO aerogel was prepared via hydrothermal reduction and freeze-drying (Figure 9c). ZnO particles were homogeneously loaded on the porous GO network. The RL_min_ of the aerogel at 2.49 mm was −79.1 dB, and the EABmax was 7.7 GHz at 1.97 mm with 20.0 wt% of filling. Furthermore, the aerogel also exhibited compression resistance and flame-retardancy [82]. A metal–organic framework-rGO (Fe-MOF-rGO) was fabricated via the solvothermal approach. With 25 wt% of Fe-MOF-rGO, the RL_min_ of the modified PW at 2 mm was −43.6 dB, and the EAB exceeded 5.0 GHz. The conduction loss of rGO, magnetic loss of Fe, and interface polarization loss of the hetero-structure contributed to the EM absorbing performance. With 10 wt% of Fe-MOF-rGO, the PHRR of the modified epoxy resin decreased by 42.3% [85].

### 4.4. High Adsorption Capacity

Oil spills, hazardous chemicals leakage, and industrial emissions have a significant influence on both the natural environment and human life. High-performance absorbents have attracted wide attention. The ideal adsorbent material should have a robust porous structure, low density, hydrophobic and oleophilic properties, and reusability. Sponges and aerogels, based on 3D graphene and its derivatives, with high absorption efficiency and flame-retardancy were explored to fulfill the above requirements [88,105]. 

**Figure 10:**
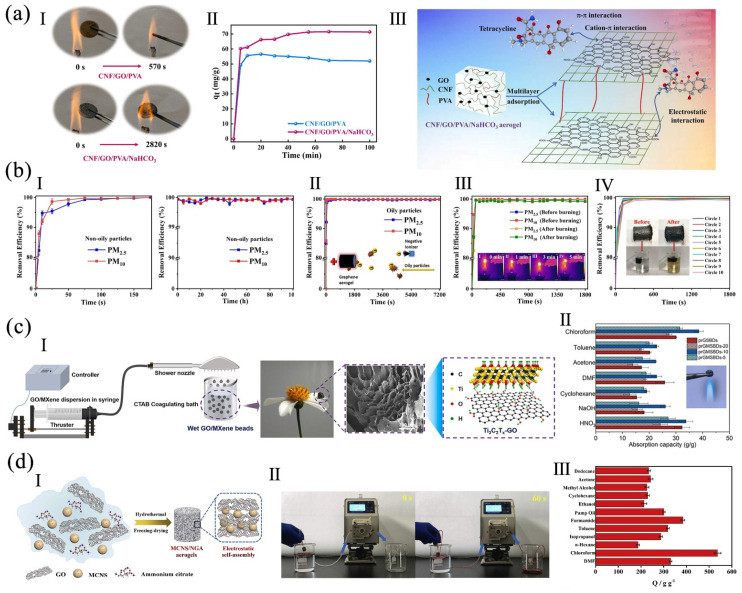
(**a**) CNF/GO/PVA/NaHCO_3_ aerogel: (**I**) aerogels during combustion, (**II**) the tetracycline adsorption capacity, (**III**) the adsorption mechanism, reproduced with permission from [106], Elsevier, 2023. (**b**) CGAF: (**I**) removal efficiency for non-oily and (**II**) oily particles, (**III**) removal efficiency before and after burning, (**IV**) cycling performance of CGAF, reproduced with permission from [107], Elsevier, 2020. (**c**) GO/MXene spherical beads: (**I**) the fabrication method, (**II**) absorption capacity, reproduced with permission from [105], Elsevier, 2022. (**d**) MCNS/NGA aerogel: (**I**) the fabrication method, (**II**) a continuous separation system for oil/water mixture, (**III**) adsorption efficiency, reproduced with permission from [17], Elsevier, 2020.

A polyphosphoric acid-modified MMT (PMMT)/rGO was prepared via hydrothermal reaction, then a PVA/PMMT/rGO aerogel was fabricated by freeze-drying. The CO_2_ adsorption capacity of PMMT/rGO and PVA/PMMT/rGO aerogels were 0.53 mmol g^−1^ and 0.238 mmol g^−1^, respectively, at 900 mmHg at 25 °C [26]. A polyvinylidene fluoride (PVDF)/SiO_2_@GO aerogel was fabricated via electrospinning and freeze-drying. With 1 wt% of SiO_2_, the aerogel had better mechanical properties, flame-retardancy, hydrophobicity, chemical resistance, and oil absorption capacity. The chloroform–water separation flux was 42,402 L m^−2^ h^−1^ with a separation efficiency of 99.96%. The absorption capacity of the aerogel to oil was 149 g g^−1^ after being reused for 10 cycles. Because of its photothermal features, it could remove thick crude oil [90]. PVA, CNF, GO, and sodium bicarbonate (NaHCO_3_) were combined by freeze-drying. Compared to CNF/GO/PVA aerogel, the thermal stability and thermal insulation of the CNF/GO/PVA/NaHCO_3_ aerogel were nearly 5 times and 3.8 times higher. The obtained aerogel had a removal ability of antibiotic tetracycline (Figure 10a), with a 71.346 mg g^−1^ adsorption capacity after 55 min of contact [106].

The space charge was introduced when the aerogel was prepared with a filter. Particulate matter was driven to the GA filter by the corona discharge (CGAF) due to the applied electric force. It had high filtering effectiveness for non-oily (>99.9%) and oily particles (>99.6%), and a low pressure drop under a high inhalable particulate matter with diameters of 2.5 μm and smaller (PM2.5) concentration of 10,000 μg m^−3^ (Figure 10b). It was fire resistant and reusable, which enabled it to burn for 5 min and 10 washings [107]. Amphipathic and porous spherical beads were fabricated from GO and MXene (Ti_3_C_2_T_x_) via self-assembly, freeze-drying, and heat treatment with a cetyltrimethylammonium bromide (CTAB) coagulation bath (Figure 10c). The beads had thermal stability and flame-retardancy to prevent ignition. The beads exhibited the fast absorption of water-based strong acid and alkali, as well as organic solvents. After five cycles, the absorption remained stable [105]. By employing alkaline ammonium citrate as a Ni source and a reducing agent, a Ni-doped magnetic carbon nanospheres/graphene (MCNS/NGA) aerogel was fabricated via hydrothermal reaction (Figure 10d). The aerogel had super-elasticity with 95% compression, flame-retardancy, and magnetism. Its adsorption capability ranged from 187 to 537 g g^−1^ for different organic solvents and oils, and it was also easily regenerable [17].

In summary, porous flame-retardant materials containing graphene with specific properties such as multiple energy conversion properties, thermal insulation, electromagnetic shielding, and high adsorption were achieved by researchers. The preparation methods, comprehensive properties, and functionalization mechanisms of these materials were proposed. How to transform the modification, dispersion, and preparation methods used in the above experiments into methods that can be mass-produced in industries while maintaining their excellent performance is a worthwhile research direction in the future.

## 5. Conclusions and Perspectives

Considerable effort has been made to develop porous materials containing graphene and its derivatives that possess both flame-retardancy and other multifunctionalities. Feasible preparation methods, including self-assembly, freeze-drying, thermal annealing, hot-pressing, spray-drying, the sol–gel method, electrospinning, electrostatic adsorption, hydrothermal reduction, in situ polymerization, vacuum-assisted impregnation, template-assisted preparation, laser scribing, etc., were explored to fabricate advanced graphene-based foams, sponges, and aerogels.

If graphene-based flame-retardant porous materials have multiple energy conversion properties, they are potential candidates for firefighter protective garments, real-time alarm sensors, electronic skin, solar energy storage, pulse monitoring, human motion detection, disaster response robots, voice recognition, and pressure sensor arrays. If they have high adsorption capacities, they are idea for handling indoor air pollution, oil–water separation, and crude oil and organics absorption. Graphene-based flame-retardant porous materials can be used in energy-efficient buildings and chemical tank transportation if they have thermal insulation properties, and can be used in stealth aircrafts and satellites if they have EM shielding properties.

The stacking and irreversible agglomeration of graphene layers led to a reduced specific surface area, less active sites, and poor regeneration, which affects their functionality. Strategies that can suppress the agglomeration and stacking of graphene, and construct optimal hierarchical porous structures, need to be studied. Moreover, many graphene-based porous materials are assembled by π–π stacking, electrostatic interactions, and H-bonding, exhibit unsatisfactory mechanical properties, and exhibit a tendency of the porous structure to deformation or collapse. Heteroatoms were doped into graphene to improve its structure stability, electrochemically active sites, and thermal and electrical conductivity. Organic–inorganic hybridization is another feasible method to improve the mechanical property and hierarchical pore structure of graphene-based materials. However, traditional doping and hybridization methods are complex, and new methods with a low cost and easy processing are required.

In practical applications, materials often need to have multiple functions and strong mechanical properties. However, the integration of strong mechanical properties and multifunctionality remains a challenge. For example, traditionally, incorporated flame retardants would mean sacrificing elasticity and energy conversion. It is necessary to understand the key factors that govern porous networks, microstructures, and mechanisms in order to achieve their functional performance. Moreover, how to balance the effect of various functions and the relationship between structure construction and the multifunctionality of GNS-based materials, is also a key challenge.
